# Orchid Root Associated Bacteria: Linchpins or Accessories?

**DOI:** 10.3389/fpls.2021.661966

**Published:** 2021-06-24

**Authors:** Jaspreet Kaur, Jyotsna Sharma

**Affiliations:** Department of Plant and Soil Science, Texas Tech University, Lubbock, TX, United States

**Keywords:** mycorrhizal fungi, mycorrhization helper bacteria, orchids, plant growth promoting rhizobacteria, rhizosphere, roots

## Abstract

Besides the plant-fungus symbiosis in arbuscular mycorrhizal (AM) and ectomycorrhizal (EM) plants, many endorhizal and rhizosphere bacteria (Root Associated Bacteria, or RAB) also enhance plant fitness, diversity, and coexistence among plants via bi- or tripartite interactions with plant hosts and mycorrhizal fungi. Assuming that bacterial associations are just as important for the obligate mycorrhizal plant family Orchidaceae, surprisingly little is known about the RAB associated with orchids. Herein, we first present the current, underwhelming state of RAB research including their interactions with fungi and the influence of holobionts on plant fitness. We then delineate the need for novel investigations specifically in orchid RAB ecology, and sketch out questions and hypotheses which, when addressed, will advance plant-microbial ecology. We specifically discuss the potential effects of beneficial RAB on orchids as: (1) Plant Growth Promoting Rhizobacteria (PGPR), (2) Mycorrhization Helper Bacteria (MHB), and (3) constituents of an orchid holobiont. We further posit that a hologenomic view should be considered as a framework for addressing co-evolution of the plant host, their obligate Orchid Mycorrhizal Fungi (OMF), and orchid RAB. We conclude by discussing implications of the suggested research for conservation of orchids, their microbial partners, and their collective habitats.

## Introduction

Symbiotic association with bacteria is a fundamental condition in plants since their terrestrialization almost 400 mya. Nonetheless, we are just beginning to understand the functional mechanisms and the breadth of the ecological consequences of plant–microbial alliances (Martin et al., 2017). Among the bacterial communities that interact with plants, bacteria that colonize root compartments including the rhizosphere and intraradical regions (i.e., Root Associated Bacteria, or RAB) can regulate the functioning of plant hosts (Box 1). Plants selectively recruit RAB in response to host genotype, metabolic profiles or root exudates and/or physicochemical properties of the soil (Bulgarelli et al., 2013). Many RAB are known to enhance plant growth through nutrient acquisition (Pii et al., 2015), hormonal provisions (Tsavkelova et al., 2016), protection from pathogens (Srivastava et al., 2016) and mitigation of abiotic stresses (Tiwari et al., 2016). Collectively, the RAB that confer growth benefits to plant hosts are termed as plant growth promoting rhizobacteria (PGPR), and generally belong to the bacterial phyla Proteobacteria, Actinobacteria, Firmicutes, and Bacteroidetes (Bulgarelli et al., 2013; Santoyo et al., 2016). Within these phyla, the genera *Rhizobium*, *Bacillus*, *Pseudomonas*, and *Burkholderia* feature prominently as PGPR, and some of their demonstrated functional contributions include mediating seed germination and subsequent plant growth (Glick, 2012; Kang et al., 2012; Walia et al., 2014). Though it is no longer questionable that symbiotic associations between roots and PGPR yield multiple growth benefits for the host plants, studies on the role of PGPR or other RAB in shaping plant ecology and evolution are scarce to non-existent (García Parisi et al., 2015; Siefert et al., 2019).

**Box 1. Glossary of key terms.**

**Root Associated Bacteria (RAB)**

Bacteria that colonize the rhizosphere and / or endorhizal spaces in a host plant.

**Plant Growth Promoting Rhizobacteria (PGPR)**

A subset of Root Associated Bacteria (RAB) that are specifically known to enhance plant growth.

**Mycorrhization Helper Bacteria (MHB)**

A group of Plant Growth Promoting Rhizobacteria (PGPR) that promote mycorrhizal colonization by improving spore germination, hyphal and root branching, or by alleviating biotic and abiotic stresses.

**Orchid Mycorrhizal Fungi (OMF)**

Fungi that establish mycorrhizal associations with orchids.

Emerging literature has shown variability in the community structure of RAB assemblages across plant taxa (Yeoh et al., 2017), seasons (He et al., 2020), and habitats (Mukhtar et al., 2018). This dynamic recruitment of RAB communities based on host phylogeny and microenvironment suggests their potential role in regulating broader ecological phenomena such as local adaptations and eco-evolutionary changes in plants. As such, rhizospheric microbiomes can impart novel traits to plants that consequently alter their ontogeny (Wagner et al., 2014; Panke-Buisse et al., 2015; Lu et al., 2018). For instance, Panke-Buisse et al. (2015) showed that three genotypes of *Arabidopsis thaliana* and *Brassica rapa* exhibited delayed flowering and increased reproductive fitness when inoculated with the rhizosphere microbiome of a late-flowering ecotype of *A. thaliana*, and this study confirmed the differences in bacterial communities in the rhizosphere of early and late flowering genotypes of *A. thaliana* and *B. rapa* by 16S rDNA sequencing. This provides some diffused evidence that by affecting the reproductive traits of plant hosts, RAB could ultimately shape their evolutionary trajectories. Ultimately, evolution is also directly influenced by reproductive barriers that steer divergence among species via reproductive isolation (Baack et al., 2015). Although geography and pollination syndromes are typically implicated in driving reproductive isolation in plants, symbiotic bacteria are known to cause reproductive isolation and speciation in insects by changing the mating preferences and hybrid lethality of their hosts (Sharon et al., 2010; Brucker and Bordenstein, 2013). Whether RAB can exert similar pressures on plant reproduction is currently unknown. Similarly, while covariation in host plant phylogeny and RAB community composition has been demonstrated in multiple studies (Peiffer et al., 2013; Bouffaud et al., 2014; Yeoh et al., 2017), the scientific community is yet to identify whether and how RAB assemblages propel plant phylogenesis and evolution.

While the magnitude of RAB effects on host ontogeny, phylogeny, and evolution remain poorly investigated, mechanisms of microbial influence on individual plants, their populations, and communities are slightly better studied and understood. Plants and microbes interact via a process described as plant soil feedbacks. As plants develop, they influence physical (soil temperature, moisture, and structure) and chemical properties (root cell lysates, pH and nutrients) of the soil surrounding them (Miki, 2012). This change in physicochemical properties causes shifts in the composition of rhizospheric bacterial and fungal communities (Miki, 2012; Krishna et al., 2020; Lin et al., 2020), which in turn alter the performance and fitness of the host. Microbe-mediated plant soil feedbacks range from positive to negative whereby either can promote species coexistence and community stabilization by affecting the fitness of key host taxa (Chung and Rudgers, 2016; Siefert et al., 2019). Negative plant soil feedbacks are generally attributed to the host-specific pathogens in soil, which often restrict the dominance of individual species in a community. In contrast, positive plant soil feedbacks reduce inter- or intraspecific competition for resources such as nutrients and soil moisture, fostering coexistence of diverse host taxa by precluding competitive exclusion. Along with promoting co-existence in plant communities, plant soil feedbacks are also known to steer community succession trajectories (Koziol and Bever, 2017; Wang et al., 2019; Zhang et al., 2020). For instance, a recent study showed that the soil fungal and bacterial communities conditioned by early successional plant species had positive effects on the mid successional species whereas soils conditioned by mid successional plant species exhibited negative effects on early successional species (Zhang et al., 2020). Interestingly, most of the plant soil feedbacks studies heretofore have focused either on Arbuscular Mycorrhizal Fungi (AMF) (Semchenko et al., 2018; Chen et al., 2019; Wang et al., 2019), or fungal pathogens (Hannula et al., 2020), whereas the stabilizing effects of PGPR, or other RAB, on plant communities largely remain neglected (Siefert et al., 2019). Such investigations are warranted for RAB to reveal their contributions, and possibly the coupled contributions of fungi and RAB, to plant community succession.

In fact, it is becoming increasingly evident that plant growth, diversity, and productivity is linked to tripartite interactions among plant hosts, PGPR, and mycorrhizal fungi (García Parisi et al., 2015; Van Der Heijden et al., 2016; Wang et al., 2016). For example, PGPR can interact with fungi to provision for plants by enhancing mycorrhizal associations. This group of PGPR is identified as Mycorrhization Helper Bacteria (MHB) that are known to stimulate expansion of mycelium, enhance lateral root formation, or modify host immunity to increase fungal infection (Bonfante and Anca, 2009; Kurth et al., 2013). Further, multiple studies have shown that RAB communities in AM and ectomycorrhizal (EcM) plant hosts are closely linked to the mycorrhizal communities (Singh et al., 2008; Vestergård et al., 2008; Bonfante and Anca, 2009; Nguyen and Bruns, 2015; Marupakula et al., 2016). Although it largely remains untested, non-random assemblies of RAB and mycorrhizal fungi in roots might be attributable to the variable specificities of RAB toward mycorrhizal fungi, or to the potential functioning of hosts, bacteria, and fungi as a unit, i.e., as a holobiont, that is putatively governed by a hologenome (Rosenberg and Zilber-Rosenberg, 2016).

First introduced by Margulis (1991) to define a host and the entirety of its endosymbionts, the term holobiont now also includes exosymbionts (Rosenberg and Zilber-Rosenberg, 2018). Simultaneously, the more recently described concept of a hologenome, defined as the combined genomes of a host and its associated microbes, represents a paradigm shift in ecology and has rapidly become a topic of interest in a wide array of organisms (Rosenberg and Zilber-Rosenberg, 2016). The hologenome concept of evolution states that a holobiont with its hologenome is a distinct biological entity and an independent unit of selection for evolution (Rosenberg and Zilber-Rosenberg, 2016). The four tenets that form the basis of the hologenome concept include: (1) all organisms possess diverse microbiota (2) organisms, along with their microbes, function as distinct biological entities, (3) a microbiome is transmitted across generations, and (4) a change in microbiome structure contributes genetic variation to hologenomes (Rosenberg and Zilber-Rosenberg, 2016). These tenets also apply to plants given the diversity of microbes found in plant species and their ability to affect plant functions. Yet, hologenomic investigations in plant systems are exceedingly scarce (Hassani et al., 2018), which is increasingly seen as a compelling argument for our general inability to explain many of the complex ecological and evolutionary phenomena.

**FIGURE 1:**
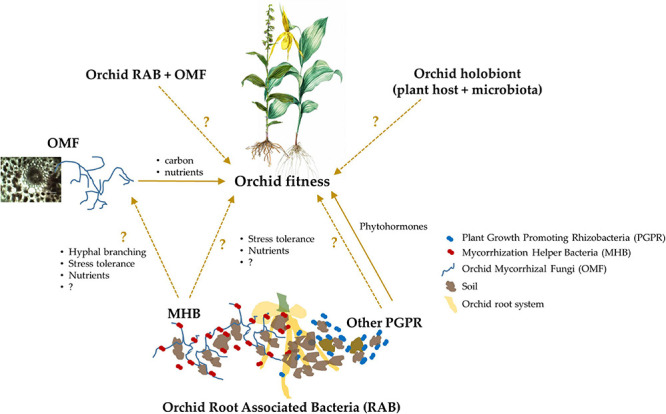
A schematic representation of the known and putative effects of orchid Root Associated Bacteria (RAB) on plant fitness, which eventually governs species ecology and evolution. Some orchid RAB are known to enhance plant fitness as Plant Growth Promoting Rhizobacteria (PGPR), and although currently unknown, they also may confer beneficial effects by helping Orchid Mycorrhizal Fungi (OMF) as Mycorrhization Helper Bacteria (MHB). The coupling of orchid RAB and OMF, and a combination of the host orchid and its microbiota (i.e., the orchid holobiont) likely also influence plant fitness, but these mechanisms are currently unknown in orchids. In the schematic, the unknown or underexplored interactions or effects are represented with dashed arrows and questions marks, while the known effects are represented with solid arrows.

While considerations of RAB and hologenomes are generally in their infancy with respect to plant ecology and evolution, their functional contributions are almost entirely neglected in one of the largest and most evolutionarily advanced plant families, the Orchidaceae. This particular group of plants represents an excellent evolutionary model system with its high diversity of taxa and ecological strategies (including pollination and mycorrhizal dependencies), unique habits, habitats, and carbon-acquisition modes across the estimated 30,000 species, and pan-global distribution. All members of the orchid family are considered rare and are afforded protection at local, regional, national, and international scales (Fay, 2018). One of the distinctive ecological requirements in all orchids is their dependence on mycorrhizal fungi that are known as Orchid Mycorrhizal Fungi (OMF) during germination and development of the rudimentary embryos. Besides, the orchid-OMF associations continue as partial or full dependence in later life stages of an orchid species depending on its photosynthetic capacity. Owing to this mycorrhizal requirement, taxonomic specificity and interactions of orchids and their OMF are generally well studied by now. Curiously though, the roles of RAB, particularly MHB, in orchid ecology have not been considered in an appreciable manner. Considering the emerging recognition of functional contributions of RAB to plant performance and evolution in other systems, it is conceivable that RAB, holobiomes, and hologenomes hold a significant unexplored potential for orchids (Figure 1). Especially considering that in a number of cases where OMF and microenvironment, either individually or together, fail to explain orchid distributions or fitness, a consideration of RAB together with OMF, environment, and holobiomes/hologenomes might allow scientists and practitioners to explain ecological and evolutionary processes in one of the largest and most distinct plant families on earth. In this review, we first present and discuss the current knowledge of orchid RAB and then present potential testable hypotheses that, when tested, will advance plant microbial ecology. The proposed conceptual models will especially inform the understanding of symbiotic interactions in one of the largest, most at-risk, and biologically complex plant families on earth.

## The Conspicuously Limited Knowledge of Orchid RAB and Their Functions

The first reports of culture-based isolations of bacteria from orchid root systems are from Australian terrestrial orchids (Wilkinson et al., 1989, 1994). Based on the fatty acid analyses, these isolates primarily belonged to the genera *Pseudomonas*, *Xanthomonas*, *Arthrobacter*, and *Bacillus* (Table 1). Similarly, based on various morphological, biochemical and / or molecular analyses, orchid RAB isolates belonging to the genera *Mycobacterium, Arthrobacter*, *Bacillus*, *Pseudomonas*, and *Rhodococcus* were identified from roots of epiphytic and terrestrial orchids in Russia (Tsavkelova et al., 2001, 2004, 2007a; Table 1). Subsequently, a study by Júnior et al. (2011) isolated bacterial strains from roots of a tropical orchid *Cattleya walkeriana*, which belonged to genera *Bacillus*, *Pseudomonas, Enterobacter*, and *Burkholderia* based on 16S rDNA sequencing. More recently, multiple studies have repeatedly reported orchid RAB belonging to the genera *Pseudomonas, Enterobacter, Sphingomonas*, and *Bacillus* from orchids in China and South America (Yang et al., 2014; Wang et al., 2016; Herrera et al., 2020).

**TABLE 1:** A summary of the genera or families of orchid Root Associated Bacteria (RAB) identified either by culture-dependent or culture-independent methods.

**Reference**	**Orchid taxon**	**Country**	**Source**	**Orchid RAB identity**
**Culture-dependent studies**				
Wilkinson et al., 1989, 1994	*Caladenia latifolia*	Australia	Root endophytes	*Pseudomonas*^†^
	*Diurnis longifolia*	Australia	Root endophytes	*Pseudomonas, Cedecea*^‡^, *Salmonella, Bacillus*^†^, *Arthrobacter*^†^, *Kurthia*^‡^, *Micrococcus*^‡^
	*Diurnis purdiei*	Australia	Root endophytes	*Pseudomonas, Bacillus*
	*Eriochilus dilatatus*	Australia	Root endophytes	*Bacillus, Kurthia, Micrococcus, Staphylococcus*^‡^
	*Leporella fimbriata*	Australia	Root endophytes	*Pseudomonas, Bacillus, Arthrobacter, Kurthia*
	*Lyperanthus nigricans*	Australia	Root endophytes	*Pseudomonas, Bacillus, Arthrobacter, Kurthia*
	*Pterostylis recurva*	Australia	Root endophytes	*Pseudomonas, Xanthomonas, Morganella*^‡^, *Acinetobacter, Bacillus, Kurthia*
	*Pterostylis vittata*	Australia	Root endophytes	*Pseudomonas, Erwinia*^‡^, *Bacillus, Kurthia*
	*Rhizanthella gardneri*	Australia	Root endophytes	*Pseudomonas, Yersinia*
	*Spiculaea ciliata*	Australia	Root endophytes	*Pseudomonas*
	*Thelymitra crinita*	Australia	Root endophytes	*Pseudomonas, Bacillus, Arthrobacter, Kurthia*
	*Thelymitra fuscolutea*	Australia	Root endophytes	*Xanthomonas*^†^, *Kurthia, Staphylococcus*
Tsavkelova et al., 2001	*Calanthe vestita var. rubrooculata*	Russia	Rhizoplane	*Pseudomonas, Bacillus, Mycobacterium*^†^, *Arthrobacter*
	*Dendrobium moschatum*	Russia	Rhizoplane	*Pseudomonas, Bacillus, Curtobacterium*^‡^, *Flavobacterium*^‡^, *Nocardia*^‡^, *Rhodococcus*^‡^, *Xanthomonas*
Tsavkelova et al., 2004	*Acampe papillosa*	Russia	Rhizoplane	*Pseudomonas, Bacillus, Flavobacterium, Rhodococcus, Micrococcus, Streptomyces*^‡^, *Cellulomonas*^‡^, *Xanthomonas, Acinetobacter, Gluconobacter, Mycobacterium*
	*Acampe papillosa*	Russia	Endorhizal	*Pseudomonas, Bacillus, Flavobacterium, Rhodococcus, Xanthomonas*
	*Dendrobium moschatum*	Russia	Rhizoplane	*Pseudomonas, Bacillus, Acinetobacter, Aquaspirillum, Rhodococcus*
Tsavkelova et al., 2007a	*Paphiopedilum appletonianum*	Vietnam	Rhizoplane	*Pseudomonas, Bacillus, Burkholderia*^‡^, *Streptomyces, Erwinia, Nocardia*
	*Paphiopedilum appletonianum*	Vietnam	Endorhizal	*Pseudomonas, Bacillus, Streptomyces, Erwinia*
	*Pholidota articulata*	Vietnam	Rhizoplane	*Pseudomonas, Bacillus, Burkholderia, Erwinia, Flavobacterium, Stenotrophomonas*^†^, *Pantoea*^‡^, *Chryseobacterium*^‡^, *Agrobacterium*^‡^, *Paracoccus*^‡^
	*Pholidota articulata*	Vietnam	Endorhizal	*Pseudomonas, Bacillus, Flavobacterium*
Júnior et al., 2011	*Cattleya walkeriana*	Brazil	Rhizoplane	*Pseudomonas, Bacillus, Burkholderia, Pantoea, Curobacterium, Enterobacter*^†^, *Achromobacter*^‡^
	*Cattleya walkeriana*	Brazil	Endorhizal	*Pseudomonas, Bacillus, Burkholderia, Pantoea, Curobacterium, Enterobacter*
Yang et al., 2014	*Dendrobium officinale*	China	Endorhizal	*Sphingomonas*^†^
Wang et al., 2016	*Dendrobium catenatum*	China	Endorhizal	*Enterobacter, Herbaspirillum*^†^
Gontijo et al., 2018	*Cymbidium* sp.	Brazil	Endorhizal	*Pseudomonas, Bacillus, Herbaspirillum, Rhizobium*^†^, *Stenotrophomonas*
Herrera et al., 2020	*Chloraea barbata*	Chile	Endorhizal	*Pseudomonas*
	*Chloraea collicensis*	Chile	Endorhizal	*Bacillus, Exiguobacterium*^‡^, *Dyella, Luteibacter*^‡^
	*Chloraea gavilu*	Chile	Endorhizal	*Pandoraea*^‡^
	*Chloraea magellanica*	Chile	Endorhizal	*Pseudomonas, Collimonas*^‡^, *Chryseobacterium*
	*Gavilea araucana*	Chile	Endorhizal	*Pseudomonas*
	*Gavilea lutea*	Chile	Endorhizal	*Pseudomonas*
**Culture-independent studies**				
Yu et al., 2013	*Dendrobium officinale*	China	Endorhizal	*Burkholderia, Rhodanobacter*^‡^, *Pseudomonas, Sphingomonas*
Li et al., 2017	*Dendrobium catenatum*	China	Endorhizal	*Pseudomonas, Delftia^‡^, *Shigella, Burkholderia, Pantoea, Sphingomonas, Buttiauxella*^‡^, *Duganella*^‡^, *Acinetobacter*^‡^, *Pectobacterium*
Novotná and Suárez, 2018	*Stanhopea connata*	Ecuador	Bacteria associated with OMF	*Bacillus, Gemella, Staphylococcus, Streptococcus, Paracoccus, Achromobacter*
Alibrandi et al., 2020	*Neottia ovata*	Italy	Endorhizal	*Cutibacterium, Rhodanobacter, Dolosigranulum, Corynebacterium
	*Serapias vomarecea*	Italy	Endorhizal	*Cutibacterium, Corynebacterium, Pseudomonas, Luteibacter
	*Spiranthes spiralis*	Italy	Endorhizal	*Mycobacterium, Candidatus Ovatusbacter
Lin et al., 2020	*Gymnadenia*	China	Endorhizal	*Comamonadaceae, Chitinophagaceae, Sphingobacteriaceae, Cytophagaceae, Rhizobaceae, Acidobacteriaceae
	*conopsea*			

Data are first organized chronologically within each of the two methodological categories, followed by an alphabetical listing of orchid taxa nested within. The country where the host orchids were sampled, source of bacteria, and the taxonomic identity are listed thereafter. Culture-dependent identification of orchid RAB was based either on the 16S rDNA barcode sequences alone, or morphological and biochemical assays alone, or a combination of the two approaches. Culture-independent methods utilized either Sanger or high throughput sequencing of the 16S rDNA bacterial barcoding region. Bacterial genera that have shown plant growth promoting activity in orchid taxa are indicated by the superscript ^†^, while the bacterial genera that have shown plant growth promoting activity in non-orchid taxa are indicated by the symbol ^‡^. *Only the dominant bacterial taxa that showed high relative abundances are listed here.

Reports of orchid RAB based on culture-independent methods are scarcer still (Table 1). Only five such studies are available, of which two utilized Sanger sequencing (Yu et al., 2013; Novotná and Suárez, 2018) and the other three utilized high throughput sequencing to sequence the 16S rDNA barcoding region of bacterial communities directly from root or OMF DNAs (Li et al., 2017; Alibrandi et al., 2020; Lin et al., 2020). The study by Yu et al. (2013) amplified endophytic bacteria belonging to *Burkholderia, Rhodanobacter, Pseudomonas* and *Sphingomonas* from the roots of *Dendrobium officinale* while Novotná and Suárez (2018) identified several bacterial strains representing genera *Bacillus*, *Streptococcus*, and *Acidobacter* associated with an orchid mycorrhizal fungus [a *Serendipita* sp.] of a tropical epiphytic orchid *Stanhopea connata*. On the other hand, Li et al. (2017) reported taxa within *Pseudomonas, Shigella, Delftia*, *Burkholderia*, *Sphingomonas*, and *Pantoea* dominating the endophytic bacterial communities in roots of an epiphytic orchid *Dendrobium catenatum* in China while Alibrandi et al. (2020) reported *Cutibacterium*, *Luteibacter*, and *Corynebacterium* as the dominant taxa from three terrestrial orchids of Italy. The third report utilizing high throughput sequencing documented the dominance of endophytic bacterial families Comamonadaceae, Chitinophagaceae, Cytophagaceae, and Sphingobacteriaceae in roots of an Asian orchid *Gymnadenia conopsea* (Lin et al., 2020). We have provided a complete list of bacteria isolated from orchid root systems in Table 1. Interestingly, orchid RAB reported so far show a remarkable overlap with bacteria commonly known as PGPR, or more specifically as MHB (Frey-Klett et al., 2007; Glick, 2012). This trend suggests that orchid RAB likely exert similar, and other as yet unknown, influences on orchids (Figure 1). We discuss below the few known, and the many potential, but yet untested, pathways through which orchid RAB can underpin orchid ecology and evolution but remain obscure. Toward this end, we first present the known roles of RAB in plant fitness and then use this information to provide similar and novel perspectives on the role of orchid RAB in orchid ecology.

## Functional Roles of RAB in Plant Biology and Perspectives on the Role of Orchid RAB in Orchid Niches

### RAB as PGPR

#### Abiotic and Biotic Stress Alleviation by Acting as Bio-Ameliorators

Plant cells perceive abiotic stresses such as freezing temperatures through the production of Reactive Oxygen Species (ROS) and calcium spiking in cytosol whereby high levels of ROS degrade nucleic acids, proteins and lipids, leading to physical damage of plant cells (Liu et al., 2017). RAB are known to mediate abiotic stress tolerance in plants by modulating the production of ROS. For instance, endophytic *Pseudomonas* strains conferred cold resistance in tomato plants through production of antioxidant enzymes that maintain ROS homeostasis in cytosol (Ding et al., 2011; Subramanian et al., 2015). Similarly, a study by Sheibani-Tezerji et al. (2015) showed that an endophytic *Burkholderia* strain was able to sense the drought stress and change its own transcriptome profile to upregulate genes associated with maintaining cellular homeostasis to destroy excessive ROS, resulting in improved plant performance under drought stress. Beneficial RAB also alleviate plant biotic stresses either directly or indirectly (Liu et al., 2017). Direct beneficial effects may be exerted by producing allelochemicals such as antibiotics, hydrogen cyanide, iron chelating siderophores or volatile organic compounds to increase resistance to plant pathogens and pests (Liu et al., 2017). Alternatively, RAB may also inhibit pathogens through the quenching mechanism that destroys the quorum sensing signals in pathogenic microorganisms (Kusari et al., 2014). Besides, endophytic RAB can inhibit pathogens indirectly by inducing systemic resistance in plants through jasmonic acid, salicylic acid, or ethylene dependent pathways and consequently priming plants for more intense defense responses (Brock et al., 2013; Glaeser et al., 2016).

#### Production of 1-Aminocyclopropane-1-Carboxylate (ACC) Deaminase

Biotic and abiotic stresses induce ethylene production in plants, which limits plant growth and enhances cell death at high concentrations (Afzal et al., 2019). Under stressful conditions, plants produce ACC (an immediate precursor to ethylene) in roots, from where it is transported to shoots and gets converted to ethylene in leaves (Liu et al., 2017). Beneficial RAB sequester ACC in roots, hydrolyze it by producing ACC deaminase and then use the byproducts as nitrogen and carbon sources (Sun et al., 2009; Glick, 2014), leading to lower ethylene levels and enhanced plant growth under stressed conditions. This effect was shown by Qin et al. (2014) when root endophytic bacteria capable of producing ACC-deaminase enhanced seed germination and plant growth in a halophyte host *Limonium sinense* under salt stress.

#### Production of Phytohormones

Genomes of RAB often possess genes encoding for proteins involved in the biosynthesis of phytohormones such as indole acetic acid (Zhang et al., 2019) and gibberellins (Nett et al., 2020). Indeed, multiple studies have corroborated the production of phytohormones in RAB (Tsavkelova et al., 2007a; Hoffman et al., 2013; Toumatia et al., 2016) and their growth enhancing effects in plants. A large body of literature has illustrated the role of bacterial indole acetic acid in increasing root biomass, surface area, and lateral branching (Patten and Glick, 2002; Tsavkelova et al., 2007a). Gibberellins produced by RAB has been shown to enhance shoot growth and chlorophyll content under abiotic stresses (Kang et al., 2014; Khan et al., 2014), and similarly RAB cytokinins improve root and shoot biomass under stressed conditions (Arkhipova et al., 2005; Liu et al., 2013).

#### Nutrient Acquisition

Nutritional provisioning by increasing the availability of nitrogen, iron and phosphorus is another way some RAB can directly promote plant growth. These elements are key components of proteins, enzymes, and other cellular compounds that drive plant physiological processes such as transpiration and respiration. Often, they are present in organic or inorganic insoluble forms that cannot be utilized by plants, and RAB can mediate their uptake. For example, RAB support the uptake of iron by producing siderophores or chelating agents that bind to the insoluble ferric ions in soil. Plants then acquire iron from bacterial siderophores through root-mediated degradation of siderophores (Rajkumar et al., 2009). Likewise, some beneficial RAB improve the availability of phosphorus by solubilizing phosphorus through acidification, chelation, or by releasing phosphatases. Besides, RAB also enhance the availability of nitrogen to plants by fixing atmospheric nitrogen through nitrogenase activity. In fact, along with the well-known legume-*Rhizobium* symbioses for nitrogen fixation, bacteria within the genera *Azospirillum, Burkholderia, Gluconacetobacter*, and *Paenibacillus* also fix atmospheric nitrogen and enhance plant biomass under nitrogen limited environments (Scherling et al., 2009; Gupta et al., 2013; Afzal et al., 2019).

Apart from promoting the growth of individual plants, PGPR are also known to enhance coexistence among plant species and thus stabilize community structures via plant soil feedback mechanisms. For instance, Siefert et al. (2019) have shown the stabilization effects of PGPR (*Rhizobium* sp.) in experimental communities where four *Trifolium* species were sown together at variable relative frequencies and inoculated with strains of *Rhizobium* isolated either from conspecific or congeneric plant hosts. The results showed that inoculation with strains from conspecific hosts reduced the fitness of *Trifolium* taxa in a frequency-dependent manner, indicating that species-specific mutualists provide negative plant soil feedback to impose self-limitation on hosts, resulting in stabilization of plant communities. In another study, Van Der Heijden et al. (2016) established gnotobiotic microcosms simulating European grasslands and demonstrated that co-inoculations of *Rhizobium* strains and mycorrhizal fungi resulted in higher plant diversity, seedling recruitment and enhanced nutrient uptake in comparison to single symbiont inoculations. This study suggested that bacteria and mycorrhizal fungi complement each other in enhancing community diversity and productivity.

### Orchid RAB as PGPR

Explaining many aspects of orchid ecology and evolution including patterns of seed germination, vegetative dormancies (i.e., absence of plant emergence or aboveground growth from perennating structures) in geophytes, or species distributions, however, has always been an enigmatic task, especially when mycorrhizal fungi and/or abiotic environment have not been entirely successful in predicting the triggers and outcomes of these processes (McCormick et al., 2016; Calevo et al., 2020). Considering the growth enhancing roles of beneficial RAB, it is highly likely that orchid RAB include beneficial bacteria that facilitate plant growth, fitness, and a number of other ecological or evolutionary processes in orchids (Figure 1). For instance, McCormick et al. (2016) reported a lack of orchid seed germination at locations away from conspecific plants even though the species-specific OMF was present, while germination was higher at locations near conspecific plants in the presence of OMF. These observations convincingly reveal that factors other than OMF steer orchid seed germination. As such, it is possible that the growth promoting activity of orchid RAB could be contributing to the variation in seed germination responses, and hence plant distributions, *in situ*.

In fact, the limited amount of literature available on orchid RAB suggests that orchid RAB can supplement orchid niches through phytohormone production, nutritional support, or by enhancing stress tolerance. The potential of several orchid RAB to produce indole acetic acid and improve seed germination and plant growth has been demonstrated by several researchers (Wilkinson et al., 1989, 1994; Tsavkelova et al., 2007b, 2016; Júnior et al., 2011; Yang et al., 2014) and hypothesized to occur *in vivo* (Teixeira da Silva et al., 2015). For example, bacterial isolates from roots of multiple orchid taxa produced indole acetic acid and enhanced mycorrhiza-assisted germination of *Pterostylis vittata* seeds (Wilkinson et al., 1989, 1994). In another study, Tsavkelova et al. (2007b) showed that bacteria isolated from the rhizoplane of *Dendrobium moschatum* produced indole acetic acid and promoted asymbiotic seed germination. More recently, Júnior et al. (2011) showed that endophytic and rhizoplane bacteria isolated from *Cattleya walkeriana* produced indole acetic acid and promoted the growth of asymbiotically germinated seedlings of *C. walkeriana* under greenhouse conditions. Overall though, besides a handful of studies focused on phytohormone production, other possible orchid RAB-mediated growth effects remain largely unknown in orchids. To our knowledge, only one study has reported the potential of orchid RAB to support the nutritional niche of orchids and to provide biotic stress tolerance (Yang et al., 2014). Therein, an endophytic bacterium *Sphingomonas paucimobilis*, isolated from the roots of *Dendrobium officinale* exhibited the capacity to fix atmospheric nitrogen, and increased seedling growth and biomass; although, a direct relationship between nitrogen fixation and seedling growth was not presented. Nonetheless, orchid RAB-mediated nutrient uptake is a plausible scenario in orchids especially within the context of their nutritional dependencies during seed germination and in subsequent life stages. Further, Yang et al. (2014) also reported that seedlings inoculated with *S. paucimobilis* showed high concentration of salicylic acid, indole acetic acid, and abscisic acid, which possibly promote growth or provide systemic resistance against biotic stresses to the host orchid as seen in other plants.

Beyond enhancing plant growth, another intriguing functional possibility is the orchid RAB-mediated regulation of vegetative dormancy in geophytic orchids. Whether such dormancies are an adaptive or a bet-hedging trait remains unknown (Shefferson et al., 2020), though they are believed to be triggered by stresses such as drought and herbivory (Shefferson et al., 2018). Abiotic and biotic stresses may cause orchid dormancy within or across growing seasons within an individual plant when resource accumulation in perennating structures is insufficient to support emergence (Shefferson et al., 2018) even when the stresses dissipate. Within this context, it is reasonable to hypothesize that orchid RAB might prevent the onset of dormancy via at least two mechanisms: (1) by providing nutritional and hormonal support to improve carbon accumulation, and/or (2) by secreting ACC deaminase and gibberellins to counteract the activity of the stress hormones ethylene and abscisic acid. Further, it is possible that orchids derive such benefits by utilizing a synergistic coupling of orchid RAB and OMF; hence, their combined contributions to orchid fitness merit just as much attention.

### RAB as MHB

While orchid RAB may directly influence orchid fitness, it is equally plausible that complementation or synergies between orchid RAB and OMF mediate the fitness of their hosts, thereby directing the fate of individuals, their populations, and the species. The concept of mycorrhization helper bacteria (MHB) was first discussed by Garbaye (1994) who defined MHB as the bacteria that promote formation of mycorrhizae. As such, MHB can be considered a subset of PGPR that may reside intimately with mycorrhizal fungi in the hyphosphere, mycorrhizosphere, or sporocarps or in other environments like plant endosphere, rhizosphere, and soil that are not directly related to fungal tissues (Deveau and Labbé, 2017). In fact, MHB are implicated in improving mycorrhizal colonization by enhancing spore germination, mycelial extension and by increasing the receptivity of roots to fungi by increasing root surface area (Frey-Klett et al., 2007). For example, Labbé et al. (2014) showed that 17 of the 21 *Pseudomonas* strains isolated from the rhizosphere or endosphere of *Populus deltoides* increased radial growth of *Laccaria bicolor* in fungal-bacterial co-cultures. Some potential mechanisms through which MHB can improve fungal growth include production of compounds such as branching factors that increase hyphal extension by changing the actin cytoskeleton of fungal hyphae (Frey-Klett et al., 2007; Schrey et al., 2007) or through nutritional complementation with vitamins or compounds that are not synthesized by mycorrhizal fungi (Hildebrandt et al., 2006; Deveau et al., 2010). In the study by Labbé et al. (2014), dual inoculation of rooted cuttings of three *Populus* species with *L. bicolor* and *Pseudomonas* strains increased the number of secondary roots and consequently mycorrhizal colonization. Nevertheless, the effects of MHB on root proliferation and morphology are not yet clear but could possibly be modulated through auxin homeostasis and ethylene production by MHB (Deveau and Labbé, 2017).

Other mechanisms by which MHB are known to promote mycorrhization include amelioration of stresses for better growth of fungi (Frey-Klett et al., 2007). To this effect, Hashem et al. (2016) showed that inoculation of *Acacia gerardii* with *Bacillus subtilis* enhanced AMF colonization by alleviating salt stress. As a consequence, plant biomass, nodulation, and crude protein content of *A. gerardii* increased when plants were co-inoculated with AMF and *B. subtilis* as opposed to independent inoculations with either symbiont. In this study, inoculations of *B. subtilis* alone or in combination with arbuscular mycorrhizal fungus resulted in higher uptake of N, P, K, Mg, Ca, enhanced activity of phosphatases and decrease in concentrations of Na and Cl in plant tissues that consequently resulted in improved mycorrhizal colonization and plant growth under salinity stress. While there are additional studies showing a positive effect of dual inoculations with bacteria and mycorrhizal fungi on plant growth and stress tolerance (Neeraj and Singh, 2011; Almethyeb et al., 2013; Vafadar et al., 2014), they all represent agricultural or forestry systems. Unfortunately, no literature is available to date that determines if and how MHB functioning shapes plant ecology and evolution in natural systems. It is especially likely that the ecology of partially- or fully mycoheterotrophic plants is more closely tied to MHB functions considering their near-complete dependence on mycorrhizal fungi.

### Orchid RAB as MHB

Considering that the life histories of orchids are intimately tied to their mycorrhizal fungal partners whereby OMF may regulate the fundamental niches of orchids by limiting germination, recruitment, and plant fitness (Swarts et al., 2010; Kaur et al., 2019), the potential contributions of MHB in orchid ecology are expected to be appreciable. Yet, to our knowledge, nothing is known of the identity, functions, and interactions of MHB associated with orchids and their OMF. Given the complete absence of studies on MHB-OMF interactions, some basic queries are warranted as a starting point. These might include an investigation of whether MHB associated with OMF, orchid tissues or orchid rhizosphere improve mycorrhizal colonization either during germination or otherwise. Investigations must also address whether MHB-OMF associations exhibit specificity toward each other and toward their orchid hosts. If such extreme specializations exist in the MHB-OMF-host interactions, their collective potential for explaining plant distributions could be remarkable, and could eventually explain species distributions and community compositions in the context of microbial niche partitioning. For instance, a community of four orchid taxa that utilize a same orchid mycorrhizal fungus might otherwise compete for soil nutrients or soil moisture, whereas their unique specificities toward MHB strains could facilitate access to non-overlapping nutrient pools such as different chemical forms of nitrogen (ammonium, nitrate, etc.), thereby promoting co-existence through niche differentiation and resource partitioning.

### RAB as Constituents of Holobionts and Hologenomes

Despite the relative infancy of plant-microbial frameworks in explaining functions, distributions, and evolution of plants, plants are no longer viewed as autonomous units (Vandenkoornhuyse et al., 2015). Microbes are implicated in ontogenic, physiological and evolutionarily development of plants (Wagner et al., 2014; Yang et al., 2014; Rosenberg and Zilber-Rosenberg, 2016). Yet, we are far from understanding the coupled effects of the various microbes, their interactions with environment, and even farther from considering holobionts and their hologenomes within these contexts (Vandenkoornhuyse et al., 2015). While the role of microbiota in host plant fitness is somewhat understood (Hassani et al., 2018), the mechanistic underpinnings of hologenomes in shaping plant ecology and evolution remain elusive. The limited empirical evidence of the contribution of hologenomes in evolution and speciation comes mostly from the animal kingdom. Accordingly, the hologenome acted as a reproductive barrier in the parasitoid wasp genus *Nasonia* (Brucker and Bordenstein, 2013) when hybrids generated by cross-breeding closely related lineages of *Nasonia* were inviable. In contrast, an antibiotic treatment rescued this hybrid lethality, suggesting that the gut microbiome of the *Nasonia* species prevented interspecific breeding. Similarly, Sharon et al. (2010) showed that a tetracycline treatment terminated the diet-induced mating preferences in *Drosophila melanogaster* and that these preferences were reinstated when flies were infected with microbes isolated from their guts. Hence, it is not only conceivable but very likely that similar mechanisms operate in the plant kingdom where microbiota alters reproductive or other biological processes that directly impact ecology and evolution of organisms.

### Orchid RAB as Constituents of Holobionts and Hologenomes

The hologenome concept has a particular relevance for orchids because of their obligate mycoheterotrophic habit during seed germination and the continued dependency on microbes throughout their life cycle depending on their trophic mode. Simultaneously, questions related to the often puzzling distribution of orchids and their rapid diversification into 25,000-30,000 species should also perhaps be framed within the context of hologenomes. As such, Givnish et al. (2015) ascribed the radiation in orchids to the evolution of pollinia, epiphytism, crassulacean acid metabolism photosynthesis, deceit pollination and tropical distributions, while leaving out OMF by citing a lack of relevant information. Not surprisingly, bacterial preferences, microbiomes, or hologenomes were not even mentioned as potential predictors in their study. Nevertheless, a few researchers have attempted to link OMF to diversification in photosynthetic orchids (Roche et al., 2010; Waterman et al., 2011; Suárez et al., 2016), suggesting that OMF do not, as yet, explain speciation within orchid lineages. Notably, with the exception of Waterman et al. (2011), each of the other two studies was focused only on a single genus of orchids, limiting the interpretation of these results and leaving much room for continued investigations. In contrast to photosynthetic orchids, the evolution of achlorophyllous, fully mycoheterotrophic orchids and mixotrophic taxa is linked to a shift in their OMF partners (Ogura-Tsujita et al., 2012; Těšitelová et al., 2015). The establishment of new and stable symbioses with microbes requires major physiological adjustments for the host as well as their microbial symbionts, and can lead to co-evolution of both partners. For this reason, the switch from mixotrophy to complete mycoheterotrophy is often accompanied by a loss of genes related to photosynthesis and an expansion of the genes involved in digestion of fungal hyphae (Yuan et al., 2018). Although not investigated, similar genomic adaptations could be expected in the mycorrhizal fungal genomes to adapt to the endophytic lifestyle in achlorophyllous orchids. Another classic case of co-evolution is evident in the evolution of endofungal bacteria that persist within AMF and have lost the ability to live independently after undergoing genome reductions (Bonfante et al., 2019). Altogether, the current state of knowledge suggests that genome rearrangements are inevitable in the establishment of highly specialized symbioses, suggesting that a consideration of hologenomes may help explain diversification in orchids.

Besides the evolutionary implications of hologenomes, both spatial and temporal changes in the hologenomic components could help optimize the functioning of holobionts and eventually predict the distribution patterns of orchids. Orchids are represented pan-globally even though the distribution ranges of individual species range from highly endemic (Swarts et al., 2010; Pandey et al., 2013; Kaur et al., 2021) to wide ranging (Hutchings, 2010; Davis et al., 2015; Kaur et al., 2019). Regardless of their distributions, however, a large majority of orchid taxa are faced with declining trends as their local populations, which are often small, are exposed to anthropogenic or environmental threats (Swarts and Dixon, 2009; Fay, 2018). Under such increasingly stressed conditions, genetic variability in hologenomes through differential recruitment of OMF and / or orchid RAB might underpin local adaptations of the orchid holobionts. In particular, association with distinct orchid RAB might be especially beneficial in conferring hologenomic variability when host orchids exhibit extreme specificity (Swarts et al., 2010; Kaur et al., 2018, 2019; Calevo et al., 2020) in their OMF preference. Altogether, we posit that combined considerations of OMF, orchid RAB and the host orchid as holobionts and hologenomes, along with their interactions with the environment, thus, merit increasing attention in our efforts to explain the distributions, adaptations, and evolution of orchids.

## Implications of Orchid RAB for Orchid Conservation

Acknowledging the pivotal role(s) of bacteria and other microorganisms in the origin and maintenance of plants and biodiversity on the planet, we specifically propose that conservation programs employ a holistic approach that emphasizes conservation of symbiotic microbes along with their hosts and their collective habitats. In fact, we suggest that conservation programs should also adopt a holobiont perspective when designing tools and strategies to preserve and protect ecosystems and their biotic components. This approach is especially suited and necessary for the Orchidaceae that are all considered ‘rare’ in nature, represent approximately 10% of all angiosperm flora on earth (and 80% of the protected species on the Convention on International Trade in Endangered Species of Wild Fauna and Flora appendices; Fay, 2018), are obligate mycoheterotrophic during germination, and represent some of the most unique evolutionary strategies among higher plants. Yet, their bilateral or multilateral interactions with bacteria remain sorely neglected. We posit that while a deeper understanding of the role of MHB may seem more urgent with respect to orchid conservation, all endophytic and epiphytic microbes deserve consideration to inform holistic conservation that embraces the functional roles of microbes in individual plants, and their capacity to regulate plant growth, species distributions, species interactions, evolution, and ecosystem functions. While such efforts are warranted, they also require a clear understanding of the contributions of distinct organisms individually and altogether. For instance, the knowledge of the structure and function of bacterial guilds associated with orchid roots is extremely limited at present though some distinct trends are noticeable and informative. As such, mirroring the patterns of OMF availability in soil, orchid RAB also tend to have lower abundances in rhizosphere soil (Kaur et al., 2020). This observation suggests that orchid RAB likely remain restricted primarily to the perennating tubers without maintaining a significant presence outside the orchid tissues. Simultaneously, if orchid RAB primarily represent MHB, then the current OMF paradigm may need to be reconsidered entirely. In essence, it may not just be the OMF but the coupling of OMF along with their MHB that regulate orchid germination and growth.

## Conclusion

Microbes are increasingly being understood to modulate plant distributions and productivity (Van Der Heijden et al., 2008). After plants, bacteria form the largest proportion of the global biomass (Bar-On et al., 2018). Combined with their current dominance, the relatively recent understanding of their evolutionary role in respiration, photosynthesis, and terrestrialization of plants (Cheng et al., 2019) points to the fact that scientists are only beginning to understand the role of bacteria in plant biology and ecology. While the symbiosis between eukaryotes and plastids has existed for 1.5 Ga, nitrogen-fixation in symbiosis with legumes is estimated to have originated about 66 mya (Doyle, 2011; Shih and Matzke, 2013). With long evolutionary histories of intimate partnerships that have driven plant evolution, it would hardly be surprising to discover additional plant functions that are underpinned by root associated bacteria.

Altogether, the largely neglected roles of microbes, including bacteria, in modulating plant and ecosystem functions deserve a deeper understanding and consideration in conservation programs. Fortunately, technological advances are assisting such efforts by allowing scientists to profile microbial communities and functions at a more accelerated pace. We suggest that the time is upon us to holistically examine, understand, and incorporate bacteria and their communities in the science and practice of orchid ecology and conservation.

## Author Contributions

Both authors conceived the idea and wrote and reviewed the manuscript.

## Conflict of Interest

The authors declare that the research was conducted in the absence of any commercial or financial relationships that could be construed as a potential conflict of interest.

## References

[B1] Afzal I. Shinwari Z. K. Sikandar S. Shahzad S. (2019). Plant beneficial endophytic bacteria: mechanisms, diversity, host range and genetic determinants. *Microbiol. Res.* 221 36–49. 10.1016/j.micres.2019.02.001 30825940

[B2] Alibrandi P. Schnell S. Perotto S. Cardinale M. (2020). Diversity and structure of the endophytic bacterial communities associated with three terrestrial orchid species as revealed by 16S rRNA gene metabarcoding. *Front. Microbiol.* 11:3207. 10.3389/fmicb.2020.604964 33519751 PMC7839077

[B3] Almethyeb M. Ruppel S. Paulsen H. M. Vassilev N. Eichler-Löbermann B. (2013). Single and combined applications of arbuscular mycorrhizal fungi and *Enterobacter* radicincitans affect nutrient uptake of faba bean and soil biological characteristics. *Appl. Agric. Forestry Res.* 3 229–234. 10.3220/LBF-2013-229-234

[B4] Arkhipova T. N. Veselov S. U. Melentiev A. I. Martynenko E. V. Kudoyarova G. R. (2005). Ability of bacterium Bacillus subtilis to produce cytokinins and to influence the growth and endogenous hormone content of lettuce plants. *Plant Soil* 272 201–209. 10.1007/s11104-004-5047-x

[B5] Baack E. Melo M. C. Rieseberg L. H. Ortiz-Barrientos D. (2015). The origins of reproductive isolation in plants. *New Phytol.* 207 968–984. 10.1111/nph.13424 25944305

[B6] Bar-On Y. M. Phillips R. Milo R. (2018). The biomass distribution on Earth. *Proc. Natl. Acad. Sci. U.S.A.* 115 6506–6511. 10.1073/pnas.1711842115 29784790 PMC6016768

[B7] Bonfante P. Anca I. (2009). Plants, mycorrhizal fungi, and bacteria: a network of interactions. *Annu. Rev. Microbiol.* 63 363–383. 10.1146/annurev.micro.091208.073504 19514845

[B8] Bonfante P. Venice F. Lanfranco L. (2019). The mycobiota: fungi take their place between plants and bacteria. *Curr. Opin. Microbiol.* 49 18–25. 10.1016/j.mib.2019.08.004 31654910

[B9] Bouffaud M. L. Poirier M. A. Muller D. Moënne-Loccoz Y. (2014). Root microbiome relates to plant host evolution in maize and other Poaceae. *Environ. Microbiol.* 16 2804–2814. 10.1111/1462-2920.12442 24588973

[B10] Brock A. K. Berger B. Mewis I. Ruppel S. (2013). Impact of the PGPB *Enterobacter* radicincitans DSM 16656 on growth, glucosinolate profile, and immune responses of *Arabidopsis thaliana*. *Microb. Ecol.* 65 661–670. 10.1007/s00248-012-0146-3 23242136

[B11] Brucker R. M. Bordenstein S. R. (2013). The hologenomic basis of speciation: gut bacteria cause hybrid lethality in the genus Nasonia. *Science* 341 667–669. 10.1126/science.1240659 23868918

[B12] Bulgarelli D. Schlaeppi K. Spaepen S. van Themaat E. V. L. Schulze-Lefert P. (2013). Structure and functions of the bacterial microbiota of plants. *Annu. Rev. Plant Biol.* 64 807–838. 10.1146/annurev-arplant-050312-120106 23373698

[B13] Calevo J. Voyron S. Ercole E. Girlanda M. (2020). Is the distribution of two rare *Orchis sister* species limited by their main mycobiont? *Diversity* 12:262. 10.3390/D12070262

[B14] Chen L. Swenson N. G. Ji N. Mi X. Ren H. Guo L. (2019). Differential soil fungus accumulation and density dependence of trees in a subtropical forest. *Science* 366 124–128. 10.1126/science.aau1361 31604314

[B15] Cheng S. Xian W. Fu Y. Marin B. Keller J. Wu T. (2019). Genomes of subaerial Zygnematophyceae provide insights into land plant evolution. *Cell* 179 1057–1067. 10.1016/j.cell.2019.10.019 31730849

[B16] Chung Y. A. Rudgers J. A. (2016). Plant–soil feedbacks promote negative frequency dependence in the coexistence of two aridland grasses. *Proc. R. Soc. B Biol. Sci.* 283:20160608. 10.1098/rspb.2016.0608 27466448 PMC4971199

[B17] Davis B. J. Phillips R. D. Wright M. Linde C. C. Dixon K. W. (2015). Continent-wide distribution in mycorrhizal fungi: implications for the biogeography of specialized orchids. *Ann. Bot.* 116 413–421. 10.1093/aob/mcv084 26105186 PMC4549956

[B18] Deveau A. Brulé C. Palin B. Champmartin D. Rubini P. Garbaye J. (2010). Role of fungal trehalose and bacterial thiamine in the improved survival and growth of the ectomycorrhizal fungus Laccaria bicolor S238N and the helper bacterium *Pseudomonas* fluorescens BBc6R8. *Environ. Microbiol. Rep.* 2 560–568. 10.1111/j.1758-2229.2010.00145.x 23766226

[B19] Deveau A. Labbé J. (2017). “Mycorrhiza helper bacteria,” in *Molecular Mycorrhizal Symbiosis*, ed. Martin F. (Hoboken, NJ: Wiley Blackwell), 437–440.

[B20] Ding S. Huang C. L. Sheng H. M. Song C. L. Li Y. B. An L. Z. (2011). Effect of inoculation with the endophyte *Clavibacter* sp. strain Enf12 on chilling tolerance in *Chorispora bungeana*. *Physiol. Plant.* 141 141–151. 10.1111/j.1399-3054.2010.01428.x 21044086

[B21] Doyle J. J. (2011). Phylogenetic perspectives on the origins of nodulation. *Mol. Plant Microbe Interact.* 24 1289–1295. 10.1094/MPMI-05-11-0114 21995796

[B22] Fay M. F. (2018). Orchid conservation: how can we meet the challenges in the twenty-first century? *Bot. Stud.* 59:16. 10.1186/s40529-018-0232-z 29872972 PMC5988927

[B23] Frey-Klett P. Garbaye J. Tarkka M. (2007). The mycorrhiza helper bacteria revisited. *New Phytol.* 176 22–36. 10.1111/j.1469-8137.2007.02191.x 17803639

[B24] Garbaye J. (1994). Helper bacteria: a new dimension to the mycorrhizal symbiosis. *New Phytol.* 128 197–210. 10.1111/j.1469-8137.1994.tb04003.x 33874371

[B25] García Parisi P. A. Lattanzi F. A. Grimoldi A. A. Omacini M. (2015). Multi-symbiotic systems: functional implications of the coexistence of grass-endophyte and legume-rhizobia symbioses. *Oikos* 124 553–560. 10.1111/oik.01540

[B26] Givnish T. J. Spalink D. Ames M. Lyon S. P. Hunter S. J. Zuluaga A. (2015). Orchid phylogenomics and multiple drivers of their extraordinary diversification. *Proc. R. Soc. B Biol. Sci.* 282:20151553. 10.1098/rspb.2015.1553 26311671 PMC4571710

[B27] Glaeser S. P. Imani J. Alabid I. Guo H. Kumar N. Kampfer P. (2016). Non-pathogenic *Rhizobium radiobacter* F4 deploys plant beneficial activity independent of its host *Piriformospora indica*. *ISME J.* 10 871–884. 10.1038/ismej.2015.163 26495996 PMC4796927

[B28] Glick B. R. (2012). Plant growth-promoting bacteria: mechanisms and applications. *Scientifica* 2012 1–15. 10.6064/2012/963401 24278762 PMC3820493

[B29] Glick B. R. (2014). Bacteria with ACC deaminase can promote plant growth and help to feed the world. *Microbiol. Res.* 169 30–39. 10.1016/j.micres.2013.09.009 24095256

[B30] Gontijo J. B. Andrade G. V. S. Baldotto M. A. Baldotto L. E. B. (2018). Bioprospecting and selection of growth-promoting bacteria for *Cymbidium* sp. orchids. *Sci. Agric.* 75 368–374. 10.1590/1678-992x-2017-0117

[B31] Gupta G. Panwar J. Jha P. N. (2013). Natural occurrence of *Pseudomonas aeruginosa*, a dominant cultivable diazotrophic endophytic bacterium colonizing *Pennisetum glaucum* (L.) R. *Br. Appl. Soil Ecol.* 64 252–261. 10.1016/j.apsoil.2012.12.016

[B32] Hannula S. E. Ma H. K. Pérez-Jaramillo J. E. Pineda A. Bezemer T. M. (2020). Structure and ecological function of the soil microbiome affecting plant–soil feedbacks in the presence of a soil-borne pathogen. *Environ. Microbiol.* 22 660–676. 10.1111/1462-2920.14882 31788934 PMC7027455

[B33] Hashem A. Abd-Allah E. F. Alqarawi A. A. Al-Huqail A. A. Wirth S. Egamberdieva D. (2016). The interaction between arbuscular mycorrhizal fungi and endophytic bacteria enhances plant growth of *Acacia gerrardii* under salt stress. *Front. Microbiol.* 7:1089. 10.3389/fmicb.2016.01089 27486442 PMC4949997

[B34] Hassani M. A. Durán P. Hacquard S. (2018). Microbial interactions within the plant holobiont. *Microbiome* 6:58. 10.1186/s40168-018-0445-0 29587885 PMC5870681

[B35] He R. Zeng J. Zhao D. Huang R. Yu Z. Wu L. (2020). Contrasting patterns in diversity and community assembly of *Phragmites australis* root-associated bacterial communities from different seasons. *Appl. Environ. Microbiol.* 86:e00379-20. 10.1128/AEM.00379-20 32385080 PMC7357477

[B36] Herrera H. Sanhueza T. Novotná A. Charles T. C. Arriagada C. (2020). Isolation and identification of endophytic bacteria from mycorrhizal tissues of terrestrial orchids from Southern Chile. *Diversity* 12:55. 10.3390/d12020055

[B37] Hildebrandt U. Ouziad F. Marner F. J. Bothe H. (2006). The bacterium *Paenibacillus validus* stimulates growth of the arbuscular mycorrhizal fungus *Glomus intraradices* up to the formation of fertile spores. *FEMS Microbiol. Lett.* 254 258–267. 10.1111/j.1574-6968.2005.00027.x 16445754

[B38] Hoffman M. T. Gunatilaka M. K. Wijeratne K. Gunatilaka L. Arnold A. E. (2013). Endohyphal bacterium enhances production of Indole-3-Acetic Acid by a foliar fungal endophyte. *PLoS One* 8:e73132. 10.1371/journal.pone.0073132 24086270 PMC3782478

[B39] Hutchings M. J. (2010). The population biology of the early spider orchid *Ophrys sphegodes* Mill. III. demography over three decades. *J. Ecol.* 98 867–878. 10.1111/j.1365-2745.2010.01661.x

[B40] Júnior R. F. G. Pedrinho E. A. N. Castellane T. C. L. Lemos E. G. D. M. (2011). Auxin-producing bacteria isolated from the roots of *Cattleya walkeriana*, an endangered Brazilian orchid, and their role in acclimatization. *Rev. Bras. Ciência Do Solo* 35 729–737. 10.1590/S0100-06832011000300008

[B41] Kang S. M. Khan A. L. Hussain J. Ali L. Kamran M. Waqas M. (2012). Rhizonin A from *Burkholderia* sp. KCTC11096 and its growth promoting role in lettuce seed germination. *Molecules* 17 7980–7988. 10.3390/molecules17077980 22759911 PMC6268351

[B42] Kang S. M. Radhakrishnan R. Khan A. L. Kim M. J. Park J. M. Kim B. R. (2014). Gibberellin secreting rhizobacterium, *Pseudomonas* putida H-2-3 modulates the hormonal and stress physiology of soybean to improve the plant growth under saline and drought conditions. *Plant Physiol. Biochem.* 84 115–124. 10.1016/j.plaphy.2014.09.001 25270162

[B43] Kaur J. Andrews L. Sharma J. (2019). High specificity of a rare terrestrial orchid toward a rare fungus within the North American Tallgrass Prairie. *Fungal Biol.* 123 895–904. 10.1016/j.funbio.2019.09.010 31733732

[B44] Kaur J. Harder C. B. Sharma J. (2020). *Characterization of Endophytic Bacterial Communities in Congeneric Temperate Orchids with Soil, Host Phenology and Population Size as the Predictors.* Washington, DC: Ecological Society of America.

[B45] Kaur J. Phillips C. Sharma J. (2021). Host population size is linked to orchid mycorrhizal fungal communities in roots and soil, which are shaped by microenvironment. *Mycorrhiza* 31 17–30. 10.1007/s00572-020-00993-5 33113039

[B46] Kaur J. Poff K. Sharma J. (2018). A rare temperate terrestrial orchid selects similar *Tulasnella taxa* in ex situ and in situ environments. *Plant Ecol.* 219 45–55. 10.1007/s11258-017-0776-0

[B47] Khan A. L. Waqas M. Kang S. M. Al-Harrasi A. Hussain J. Al-Rawahi A. (2014). Bacterial endophyte *Sphingomonas* sp. LK11 produces gibberellins and IAA and promotes tomato plant growth. *J. Microbiol.* 52 689–695. 10.1007/s12275-014-4002-7 24994010

[B48] Koziol L. Bever J. D. (2017). The missing link in grassland restoration: arbuscular mycorrhizal fungi inoculation increases plant diversity and accelerates succession. *J. Appl. Ecol.* 54 1301–1309. 10.1111/1365-2664.12843

[B49] Krishna M. Gupta S. Delgado-Baquerizo M. Morriën E. Garkoti S. C. Chaturvedi R. (2020). Successional trajectory of bacterial communities in soil are shaped by plant-driven changes during secondary succession. *Sci. Rep.* 10:9864. 10.1038/s41598-020-66638-x 32555419 PMC7299987

[B50] Kurth F. Zeitler K. Feldhahn L. Neu T. R. Weber T. Kristufek V. (2013). Detection and quantification of a mycorrhization helper bacterium and a mycorrhizal fungus in plant-soil microcosms at different levels of complexity. *BMC Microbiol.* 13:205. 10.1186/1471-2180-13-205 24025151 PMC3848169

[B51] Kusari P. Kusari S. Lamshöft M. Sezgin S. Spiteller M. Kayser O. (2014). Quorum quenching is an antivirulence strategy employed by endophytic bacteria. *Appl. Microbiol. Biotechnol.* 98 7173–7183. 10.1007/s00253-014-5807-3 24846733

[B52] Labbé J. L. Weston D. J. Dunkirk N. Pelletier D. A. Tuskan G. A. (2014). Newly identified helper bacteria stimulate ectomycorrhizal formation in *Populus*. *Front. Plant Sci.* 5:579. 10.3389/fpls.2014.00579 25386184 PMC4208408

[B53] Li O. Xiao R. Sun L. Guan C. Kong D. Hu X. (2017). Bacterial and diazotrophic diversities of endophytes in *Dendrobium catenatum* determined through barcoded pyrosequencing. *PLoS One* 12:e0184717. 10.1371/journal.pone.0184717 28931073 PMC5607135

[B54] Lin M. Xiong H. Xiang X. Zhou Z. Liang L. Mei Z. (2020). The effect of plant geographical location and developmental stage on root-associated microbiomes of *Gymnadenia conopsea*. *Front. Microbiol.* 11:1257. 10.3389/fmicb.2020.01257 32625183 PMC7314937

[B55] Liu F. Xing S. Ma H. Du Z. Ma B. (2013). Cytokinin-producing, plant growth-promoting rhizobacteria that confer resistance to drought stress in Platycladus orientalis container seedlings. *Appl. Microbiol. Biotechnol.* 97 9155–9164. 10.1007/s00253-013-5193-2 23982328

[B56] Liu H. Carvalhais L. C. Crawford M. Singh E. Dennis P. G. Pieterse C. M. (2017). Inner plant values: diversity, colonization and benefits from endophytic bacteria. *Front. Microbiol.* 8:2552. 10.3389/fmicb.2017.02552 29312235 PMC5742157

[B57] Lu T. Ke M. Lavoie M. Jin Y. Fan X. Zhang Z. (2018). Rhizosphere microorganisms can influence the timing of plant flowering. *Microbiome* 6:231. 10.1186/s40168-018-0615-0 30587246 PMC6307273

[B58] Margulis L. (1991). “Symbiogenesis and symbionticism,” in *Symbiosis as a Source of Evolutionary Innovation: Speciation and Morphogenesis*, eds Margulis L. Fester R. (Cambridge, MA: MIT Press), 1–14. 11538111

[B59] Martin F. M. Uroz S. Barker D. G. (2017). Ancestral alliances: plant mutualistic symbioses with fungi and bacteria. *Science* 356:eaad4501. 10.1126/science.aad4501 28546156

[B60] Marupakula S. Mahmood S. Finlay R. D. (2016). Analysis of single root tip microbiomes suggests that distinctive bacterial communities are selected by *Pinus sylvestris* roots colonized by different ectomycorrhizal fungi. *Environ. Microbiol.* 18 1470–1483. 10.1111/1462-2920.13102 26521936

[B61] McCormick M. K. Taylor D. L. Whigham D. F. Burnett R. K. (2016). Germination patterns in three terrestrial orchids relate to abundance of mycorrhizal fungi. *J. Ecol.* 104 744–754. 10.1111/1365-2745.12556

[B62] Miki T. (2012). Microbe-mediated plant-soil feedback and its roles in a chnaging world. *Ecol. Res.* 27 509–520. 10.1007/s11284-012-0937-5

[B63] Mukhtar S. Mirza B. S. Mehnaz S. Mirza M. S. Mclean J. Malik K. A. (2018). Impact of soil salinity on the microbial structure of halophyte rhizosphere microbiome. *World J. Microbiol. Biotechnol.* 34 1–17. 10.1007/s11274-018-2509-5 30128756

[B64] Neeraj Singh K. (2011). Organic amendments to soil inoculated arbuscular mycorrhizal fungi and *Pseudomonas fluorescens* treatments reduce the development of root-rot disease and enhance the yield of *Phaseolus vulgaris* L. *Eur. J. Soil Biol.* 47 288–295. 10.1016/j.ejsobi.2011.07.002

[B65] Nett R. S. Nguyen H. Nagel R. Marcassa A. Charles T. C. Friedberg I. (2020). Unraveling a tangled skein: evolutionary analysis of the bacterial gibberellin biosynthetic operon. *mSphere* 5:e00292-20. 10.1128/msphere.00292-20 32493722 PMC7273348

[B66] Nguyen N. H. Bruns T. D. (2015). The microbiome of *Pinus muricata* ectomycorrhizae: community assemblages, fungal species effects, and Burkholderia as important bacteria in multipartnered symbioses. *Microb. Ecol.* 69 914–921. 10.1007/s00248-015-0574-y 25687126

[B67] Novotná A. Suárez J. P. (2018). Molecular detection of bacteria associated with Serendipita sp., a mycorrhizal fungus from the orchid *Stanhopea connata* Klotzsch in southern Ecuador. *Bot. Lett.* 165 307–313. 10.1080/23818107.2018.1436087

[B68] Ogura-Tsujita Y. Yokoyama J. Miyoshi K. Yukawa T. (2012). Shifts in mycorrhizal fungi during the evolution of autotrophy to mycoheterotrophy in Cymbidium (Orchidaceae). *Am. J. Bot.* 99 1158–1176. 10.3732/ajb.1100464 22763355

[B69] Pandey M. Sharma J. Taylor D. L. Yadon V. L. (2013). A narrowly endemic photosynthetic orchid is non-specific in its mycorrhizal associations. *Mol. Ecol.* 22 2341–2354. 10.1111/mec.12249 23432406

[B70] Panke-Buisse K. Poole A. C. Goodrich J. K. Ley R. E. Kao-Kniffin J. (2015). Selection on soil microbiomes reveals reproducible impacts on plant function. *ISME J.* 9 980–989. 10.1038/ismej.2014.196 25350154 PMC4817706

[B71] Patten C. L. Glick B. R. (2002). Role of *Pseudomonas* putida indoleacetic acid in development of the host plant root system. *Appl. Environ. Microbiol.* 68 3795–3801. 10.1128/AEM.68.8.3795-3801.2002 12147474 PMC124051

[B72] Peiffer J. A. Spor A. Koren O. Jin Z. Tringe S. G. Dangl J. L. (2013). Diversity and heritability of the maize rhizosphere microbiome under field conditions. *Proc. Natl. Acad. Sci. U.S.A.* 110 6548–6553. 10.1073/pnas.1302837110 23576752 PMC3631645

[B73] Pii Y. Mimmo T. Tomasi N. Terzano R. Cesco S. Crecchio C. (2015). Microbial interactions in the rhizosphere: beneficial influences of plant growth-promoting rhizobacteria on nutrient acquisition process. A review. *Biol. Fertil. Soils* 51 403–415. 10.1007/s00374-015-0996-1

[B74] Qin S. Zhang Y. J. Yuan B. Xu P. Y. Xing K. Wang J. (2014). Isolation of ACC deaminase-producing habitat-adapted symbiotic bacteria associated with halophyte *Limonium sinense* (Girard) Kuntze and evaluating their plant growth-promoting activity under salt stress. *Plant Soil* 374 753–766. 10.1007/s11104-013-1918-3

[B75] Rajkumar M. Ae N. Freitas H. (2009). Endophytic bacteria and their potential to enhance heavy metal phytoextraction. *Chemosphere* 77 153–160. 10.1016/j.chemosphere.2009.06.047 19647283

[B76] Roche S. A. Carter R. J. Peakall R. Smith L. M. Whitehead M. R. Linde C. C. (2010). A narrow group of monophyletic Tulasnella (Tulasnellaceae) symbiont lineages are associated with multiple species of *Chiloglottis* (Orchidaceae): implications for orchid diversity. *Am. J. Bot.* 97 1313–1327. 10.3732/ajb.1000049 21616884

[B77] Rosenberg E. Zilber-Rosenberg I. (2016). Microbes drive evolution of animals and plants: the hologenome concept. *mBio* 7:e01395-15. 10.1128/mBio.01395-15 27034283 PMC4817260

[B78] Rosenberg E. Zilber-Rosenberg I. (2018). The hologenome concept of evolution after 10 years. *Microbiome* 6:78. 10.1186/s40168-018-0457-9 29695294 PMC5922317

[B79] Santoyo G. Moreno-Hagelsieb G. del Carmen Orozco-Mosqueda M. Glick B. R. (2016). Plant growth-promoting bacterial endophytes. *Microbiol. Res.* 183 92–99. 10.1016/j.micres.2015.11.008 26805622

[B80] Scherling C. Ulrich K. Ewald D. Weckwerth W. (2009). A metabolic signature of the beneficial interaction of the endophyte *Paenibacillus* sp. isolate and in vitro-grown poplar plants revealed by metabolomics. *Mol. Plant Microbe Interact.* 22 1032–1037. 10.1094/MPMI-22-8-1032 19589078

[B81] Schrey S. D. Salo V. Raudaskoski M. Hampp R. Nehls U. Tarkka M. T. (2007). Interaction with mycorrhiza helper bacterium *Streptomyces* sp. AcH 505 modifies organisation of actin cytoskeleton in the ectomycorrhizal fungus *Amanita muscaria* (fly agaric). *Curr. Genet.* 52 77–85. 10.1007/s00294-007-0138-x 17632722

[B82] Semchenko M. Leff J. W. Lozano Y. M. Saar S. Davison J. Wilkinson A. (2018). Fungal diversity regulates plant-soil feedbacks in temperate grassland. *Sci. Adv.* 4:eaau4578. 10.1126/sciadv.aau4578 30498781 PMC6261650

[B83] Sharon G. Segal D. Ringo J. M. Hefetz A. Rosenberg I. Rosenberg E. (2010). Commensal bacteria play a role in mating preference of *Drosophila melanogaster*. *Proc. Natl. Acad. Sci. U.S.A.* 107 20051–20056. 10.1073/pnas.1009906107 21041648 PMC2993361

[B84] Shefferson R. P. Jacquemyn H. Kull T. Hutchings M. J. (2020). The demography of terrestrial orchids: life history, population dynamics and conservation. *Bot. J. Linn. Soc.* 192 315–332. 10.1093/botlinnean/boz084

[B85] Shefferson R. P. Kull T. Hutchings M. J. Selosse M. A. Jacquemyn H. Kellett K. M. (2018). Drivers of vegetative dormancy across herbaceous perennial plant species. *Ecol. Lett.* 21 724–733. 10.1111/ele.12940 29575384

[B86] Sheibani-Tezerji R. Rattei T. Sessitsch A. Trognitz F. Mitter B. (2015). Transcriptome profiling of the endophyte *Burkholderia phytofirmans* psjn indicates sensing of the plant environment and drought stress. *mBio* 6:e00621-15. 10.1128/mBio.00621-15 26350963 PMC4600099

[B87] Shih P. M. Matzke N. J. (2013). Primary endosymbiosis events date to the later Proterozoic with cross-calibrated phylogenetic dating of duplicated ATPase proteins. *Proc. Natl. Acad. Sci. U.S.A.* 110 12355–12360. 10.1073/pnas.1305813110 23776247 PMC3725117

[B88] Siefert A. Zillig K. W. Friesen M. L. Strauss S. Y. (2019). Mutualists stabilize the coexistence of congeneric legumes. *Am. Nat.* 193 200–212. 10.1086/701056 30720367

[B89] Singh B. K. Nunan N. Ridgway K. P. McNicol J. Young J. P. W. Daniell T. J. (2008). Relationship between assemblages of mycorrhizal fungi and bacteria on grass roots. *Environ. Microbiol.* 10 534–541. 10.1111/j.1462-2920.2007.01474.x 18081854

[B90] Srivastava S. Bist V. Srivastava S. Singh P. C. Trivedi P. K. Asif M. H. (2016). Unraveling aspects of *Bacillus amyloliquefaciens* mediated enhanced production of rice under biotic stress of *Rhizoctonia solani*. *Front. Plant Sci.* 7:587. 10.3389/fpls.2016.00587 27200058 PMC4858605

[B91] Suárez J. P. Eguiguren J. S. Herrera P. Jost L. (2016). Do mycorrhizal fungi drive speciation in *Teagueia* (Orchidaceae) in the upper Pastaza watershed of Ecuador? *Symbiosis* 69 161–168. 10.1007/s13199-016-0399-6

[B92] Subramanian P. Mageswari A. Kim K. Lee Y. Sa T. (2015). Psychrotolerant endophytic *Pseudomonas* sp. strains OB155 and OS261 induced chilling resistance in tomato plants (*Solanum lycopersicum* Mill.) by activation of their antioxidant capacity. *Mol. Plant Microbe Interact.* 28 1073–1081. 10.1094/MPMI-01-15-0021-R 26075827

[B93] Sun Y. Cheng Z. Glick B. R. (2009). The presence of a 1-aminocyclopropane-1-carboxylate (ACC) deaminase deletion mutation alters the physiology of the endophytic plant growth-promoting bacterium *Burkholderia phytofirmans* PsJN. *FEMS Microbiol. Lett.* 296 131–136. 10.1111/j.1574-6968.2009.01625.x 19459964

[B94] Swarts N. D. Dixon K. W. (2009). Perspectives on orchid conservation in botanic gardens. *Trends Plant Sci.* 14 590–598. 10.1016/j.tplants.2009.07.008 19733499

[B95] Swarts N. D. Sinclair E. A. Francis A. Dixon K. W. (2010). Ecological specialization in mycorrhizal symbiosis leads to rarity in an endangered orchid. *Mol. Ecol.* 19 3226–3242. 10.1111/j.1365-294X.2010.04736.x 20618899

[B96] Teixeira da Silva J. A. Tsavkelova E. A. Zeng S. Ng T. B. Parthibhan S. Dobranszki J. (2015). Symbiotic in vitro seed propagation of *Dendrobium*: fungal and bacterial partners and their influence on plant growth and development. *Planta* 242 1–22. 10.1007/s00425-015-2301-9 25940846

[B97] Těšitelová T. Kotilínek M. Jersáková J. Joly F. X. Košnar J. Tatarenko I. (2015). Two widespread green *Neottia* species (Orchidaceae) show mycorrhizal preference for Sebacinales in various habitats and ontogenetic stages. *Mol. Ecol.* 24 1122–1134. 10.1111/mec.13088 25612936

[B98] Tiwari S. Lata C. Chauhan P. S. Nautiyal C. S. (2016). *Pseudomonas* putida attunes morphophysiological, biochemical and molecular responses in *Cicer arietinum* L. during drought stress and recovery. *Plant Physiol. Biochem.* 99 108–117. 10.1016/j.plaphy.2015.11.001 26744996

[B99] Toumatia O. Compant S. Yekkour A. Goudjal Y. Sabaou N. Mathieu F. (2016). Biocontrol and plant growth promoting properties of *Streptomyces mutabilis* strain IA1 isolated from a Saharan soil on wheat seedlings and visualization of its niches of colonization. *S. Afr. J. Bot.* 105 234–239. 10.1016/j.sajb.2016.03.020

[B100] Tsavkelova E. A. Cherdyntseva T. A. Botina S. G. Netrusov A. I. (2007a). Bacteria associated with orchid roots and microbial production of auxin. *Microbiol. Res.* 162 69–76. 10.1016/j.micres.2006.07.014 17140781

[B101] Tsavkelova E. A. Cherdyntseva T. A. Klimova S. Y. Shestakov A. I. Botina S. G. Netrusov A. I. (2007b). Orchid-associated bacteria produce indole-3-acetic acid, promote seed germination, and increase their microbial yield in response to exogenous auxin. *Arch. Microbiol.* 188 655–664. 10.1007/s00203-007-0286-x 17687544

[B102] Tsavkelova E. A. Cherdyntseva T. A. Lobakova E. S. Kolomeitseva G. L. Netrusov A. I. (2001). Microbiota of the orchid rhizoplane. *Microbiology* 70 492–497. 10.1023/A:1010402715376 11558285

[B103] Tsavkelova E. A. Cherdyntseva T. A. Netrusov A. I. (2004). Bacteria associated with the roots of epiphytic orchids. *Microbiology* 73 710–715. 10.1007/s11021-005-0013-z 15688942

[B104] Tsavkelova E. A. Egorova M. A. Leontieva M. R. Malakho S. G. Kolomeitseva G. L. Netrusov A. I. (2016). Dendrobium nobile Lindl. seed germination in co-cultures with diverse associated bacteria. *Plant Growth Regul.* 80 79–91. 10.1007/s10725-016-0155-1

[B105] Vafadar F. Amooaghaie R. Otroshy M. (2014). Effects of plant-growth-promoting rhizobacteria and arbuscular mycorrhizal fungus on plant growth, stevioside, NPK, and chlorophyll content of *Stevia rebaudiana*. *J. Plant Interact.* 9 128–136. 10.1080/17429145.2013.779035

[B106] Van Der Heijden M. G. A. Bardgett R. D. Van Straalen N. M. (2008). The unseen majority: soil microbes as drivers of plant diversity and productivity in terrestrial ecosystems. *Ecol. Lett.* 11 296–310. 10.1111/j.1461-0248.2007.01139.x 18047587

[B107] Van Der Heijden M. G. A. Bruin S. D. Luckerhoff L. Van Logtestijn R. S. P. Schlaeppi K. (2016). A widespread plant-fungal-bacterial symbiosis promotes plant biodiversity, plant nutrition and seedling recruitment. *ISME J.* 10 389–399. 10.1038/ismej.2015.120 26172208 PMC4737930

[B108] Vandenkoornhuyse P. Quaiser A. Duhamel M. Le Van A. Dufresne A. (2015). The importance of the microbiome of the plant holobiont. *New Phytol.* 206 1196–1206. 10.1111/nph.13312 25655016

[B109] Vestergård M. Henry F. Rangel-Castro J. I. Michelsen A. Prosser J. I. Christensen S. (2008). Rhizosphere bacterial community composition responds to arbuscular mycorrhiza, but not to reductions in microbial activity induced by foliar cutting. *FEMS Microbiol. Ecol.* 64 78–89. 10.1111/j.1574-6941.2008.00447.x 18312375

[B110] Wagner M. R. Lundberg D. S. Coleman-Derr D. Tringe S. G. Dangl J. L. Mitchell-Olds T. (2014). Natural soil microbes alter flowering phenology and the intensity of selection on flowering time in a wild *Arabidopsis* relative. *Ecol. Lett.* 17 717–726. 10.1111/ele.12276 24698177 PMC4048358

[B111] Walia A. Mehta P. Chauhan A. Shirkot C. K. (2014). Effect of Bacillus subtilis strain CKT1 as inoculum on growth of tomato seedlings under net house conditions. *Proc. Natl. Acad. Sci. India Sect. B Biol. Sci.* 84 145–155. 10.1007/s40011-013-0189-3

[B112] Wang G. Schultz P. Tipton A. Zhang J. Zhang F. Bever J. D. (2019). Soil microbiome mediates positive plant diversity-productivity relationships in late successional grassland species. *Ecol. Lett.* 22 1221–1232. 10.1111/ele.13273 31131969

[B113] Wang X. Yam T. W. Meng Q. Zhu J. Zhang P. Wu H. (2016). The dual inoculation of endophytic fungi and bacteria promotes seedlings growth in *Dendrobium catenatum* (Orchidaceae) under in vitro culture conditions. *Plant Cell.* 126 523–531. 10.1007/s11240-016-1021-6

[B114] Waterman R. J. Bidartondo M. I. Stofberg J. Combs J. K. Gebauer G. Savolainen V. (2011). The effects of above- and belowground mutualisms on orchid speciation and coexistence. *Am. Nat.* 177 E54–E68. 10.1086/657955 21460551

[B115] Wilkinson K. G. Dixon K. W. Sivasithamparam K. (1989). Interaction of soil bateria, mycorrhizal fungi and orchid seed in relation to germination of Australian orchids. *New Phytol.* 112 429–435. 10.1111/j.1469-8137.1989.tb00334.x

[B116] Wilkinson K. G. Dixon K. W. Sivasithamparam K. Ghisalberti E. L. (1994). Effect of IAA on symbiotic germination of an Australian orchid and its production by orchid-associated bacteria. *Plant Soil* 159 291–295. 10.1007/BF00009292

[B117] Yang S. Zhang X. Cao Z. Zhao K. Wang S. Chen M. (2014). Growth-promoting *Sphingomonas paucimobilis* ZJSH1 associated with *Dendrobium officinale* through phytohormone production and nitrogen fixation. *Microb. Biotechnol.* 7 611–620. 10.1111/1751-7915.12148 25142808 PMC4265079

[B118] Yeoh Y. K. Dennis P. G. Paungfoo-Lonhienne C. Weber L. Brackin R. Ragan M. A. (2017). Evolutionary conservation of a core root microbiome across plant phyla along a tropical soil chronosequence. *Nat. Commun.* 8:215. 10.1038/s41467-017-00262-8 28790312 PMC5548757

[B119] Yu J. Zhou X. F. Yang S. J. Liu W. H. Hu X. F. (2013). Design and application of specific 16S rDNA-targeted primers for assessing endophytic diversity in *Dendrobium officinale* using nested PCR-DGGE. *Appl. Microbiol. Biotechnol.* 97 9825–9836. 10.1007/s00253-013-5294-y 24127138

[B120] Yuan Y. Jin X. Liu J. Zhao X. Zhou J. Wang X. (2018). The Gastrodia elata genome provides insights into plant adaptation to heterotrophy. *Nat. Commun.* 9:1615. 10.1038/s41467-018-03423-5 29691383 PMC5915607

[B121] Zhang J. Ai Z. Xu H. Liu H. Wang G. Deng L. (2020). Plant-microbial feedback in secondary succession of semiarid grasslands. *Sci. Total Environ.* 760:143389. 10.1016/j.scitotenv.2020.143389 33190882

[B122] Zhang P. Jin T. Sahu S. K. Xu J. Shi Q. Liu H. (2019). The distribution of tryptophan-dependent indole-3-acetic acid synthesis pathways in bacteria unraveled by large-scale genomic analysis. *Molecules* 24:1411. 10.3390/molecules24071411 30974826 PMC6479905

